# Uptake of Respiratory Syncytial Virus Prefusion F Protein Vaccination during Pregnancy and nirsevimab among Infants over Two Seasons in a Single Health System

**DOI:** 10.1016/j.jpedcp.2026.200241

**Published:** 2026-09-15

**Authors:** Jessica E. Kerr, Annaliese J. Calzadilla, Hui Liu, Anne-Marie Rick

**Affiliations:** 1 Division of General Academic Pediatrics, Department of Pediatrics, University of Pittsburgh School of Medicine, Pittsburgh; 2 University of Pittsburgh School of Medicine, Pittsburgh

**Keywords:** respiratory syncytial virus, RSVpreF, nirsevimab, immunization, infant, pregnancy

## Abstract

**Objective:**

To describe the uptake of respiratory syncytial virus (RSV) prevention products during the first 2 seasons after availability in the US in a large health system.

**Study design:**

Using multisource immunization data from a single health system, we identified a retrospective cohort of infants born from October 2023 to April 2024 (season 1) and from September 2024 to April 2025 (season 2) and their birth parents. We described the uptake of RSV prefusion F protein (RSVpreF) vaccination and nirsevimab and evaluated factors associated with receipt using multivariable logistic regression.

**Results:**

Among 10 196 infants, 5868/10 196 (57.6%) were exposed to an RSV-preventive product. Overall, in our maternal-infant dyads, 3850/10 196 (37.8% of total) received RSVpreF, 1837/10 196 infants (18.0% of total) received nirsevimab, and in 181/10 196 (1.8%), both were received. Uptake increased from 2100/4555 (46.1%) in season 1 to 3768/5641 (66.8%) in season 2 (*P* < .001). Maternal RSVpreF vaccination increased from 1280/4555 (28.1%) to 2570/5641 (45.6%), and nirsevimab uptake increased from 749/4555 (16.4%) to 1088/5641 (19.3% both *P* < .001). Nirsevimab receipt at <6 days of life increased from 242/749 (32.2%) to 502/1088 (46.1%, *P* < .001). RSVpreF uptake was higher among older, privately insured pregnant individuals and those engaged in other preventative care and lower among those with higher area deprivation, more complex obstetric histories, or other children. Nirsevimab uptake was higher among infants with prematurity or from multifetal births and lower among those with siblings, later-season birth, or no early outpatient care. Social and structural disparities in nirsevimab uptake improved in the second season, whereas disparities in RSVpreF uptake persisted.

**Conclusions:**

The uptake of RSV preventive strategies increased across 2 seasons, with a higher uptake of maternal RSVpreF vaccination than nirsevimab.

The 2023 introduction of 2 new, seasonal respiratory syncytial virus (RSV) immunization strategies in the US marked a historic shift in RSV prevention. During the 2023-2024 and 2024-2025 RSV seasons, the Center for Disease Control and Prevention's Advisory Committee on Immunization Practices recommended maternal bivalent respiratory syncytial virus prefusion F protein (RSVpreF) vaccination between 32 and 36 weeks of gestation from September to January and administration of long-acting monoclonal antibody, nirsevimab, for infants <8 months of age born or entering their first RSV season between October and March.^1^^,^^2^ Although these interventions are complementary for reducing severe RSV disease during an infant's first months of life, they differ fundamentally in their mechanisms of protection, timing of administration, delivery settings, and cost.^1^^,^^2^ Consequently, implementing these products in clinical practice presents distinct challenges and opportunities to reach different infant populations. Although the uptake of maternal RSV vaccination may be influenced by prenatal care access, obstetric provider familiarity with RSV, vaccine confidence during pregnancy, and timing of vaccination relative to delivery, the uptake of nirsevimab may depend on insurance coverage, coordination across birth hospitals and outpatient settings, and product availability, particularly during the 2023-2024 season.^3^^,^^4^ These influences may lead to differential uptake across sociodemographic, clinical, and health system contexts.

Limited data are published on the coimplementation and uptake of these 2 products in real-world US populations during the first 2 seasons after licensure.^5^^,^^6^ Understanding where uptake of these interventions converges—and where it diverges—is critical to identifying gaps in RSV prevention, informing implementation strategies, and ensuring equitable access to protection during early infancy. This is especially important in the current environment of heightened vaccine hesitancy and evolving immunization recommendations. In this study, we leverage comprehensive electronic health record (EHR) data from an integrated health care system to describe uptake of maternal RSVpreF vaccination and infant nirsevimab among infants born during the first 2 RSV seasons after product licensure in the US. We also identify demographic, clinical, and health care access factors receipt for each product. By limiting our analysis to infants born during respiratory season and thus have potential access to either product, we obtain a clearer understanding of the factors associated with receipt of each product as well as potential gaps. Furthermore, this 2-season analysis provides insight into how patterns of uptake are changing as early implementation challenges are resolved and acceptance evolves.

## Methods

### Study Population

We used EHR data to identify a retrospective cohort of pregnant individuals who delivered infants from October 1, 2023, to April 15, 2024 (season 1) and from September 1, 2024, to April 30, 2025 (season 2) at 2 large birthing hospitals within the University of Pittsburgh Medical Center health system in western Pennsylvania. Dates were based on local RSV product availability and recommended administration windows. Infants were excluded if they were born before 32 weeks of gestational age, if they were born stillborn or had death on the day of birth, if the birth parent was ≥37 weeks of gestation at the study entry date, or if the birth parent had no prenatal care during pregnancy in the health system by 32 weeks of gestation (Figure 1). This study was approved by the University of Pittsburgh Institutional Review Board (STUDY23090170) under a waiver of consent and is reported following STROBE guidelines.^7^

**Figure 1:**
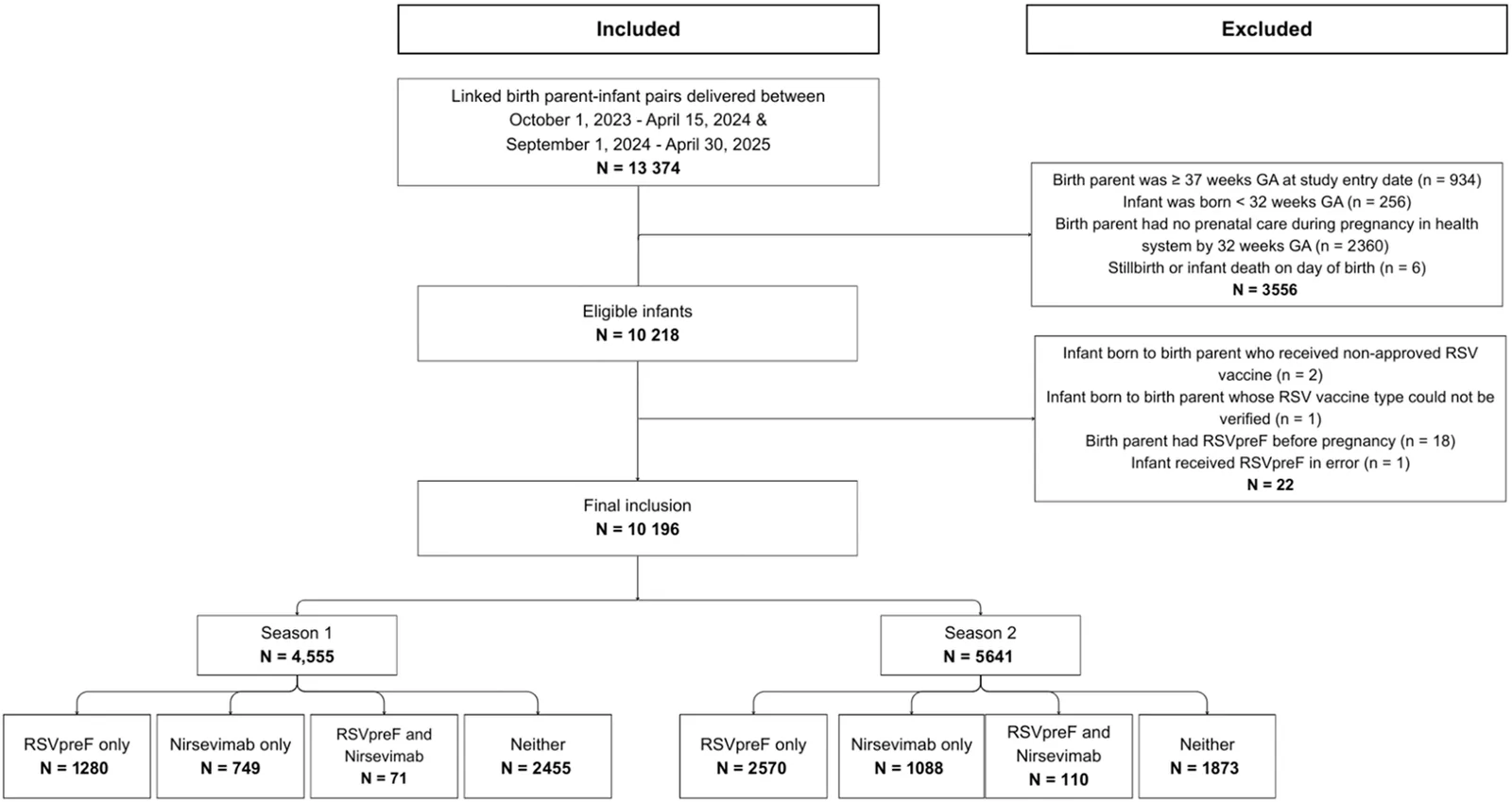
Flowchart of inclusion and exclusion criteria for population of both seasons. Selection criteria for analysis population broken down by season and receipt or not of RSV product and type.

### Data Sources

Structured data containing maternal and infant *International Classification of Diseases, 10 Revision*, diagnosis and procedure codes, immunizations, medications, prescriptions, and delivery characteristics occurring during pregnancy from time periods defined previously were obtained from institutional databases validated across EHR platforms from inpatient and outpatient settings.^8^^,^^9^ Maternal vaccination and infant receipt of nirsevimab for the cohort were also obtained through the Pennsylvania Immunization Electronic Registry System (PIERS). Data validation and data discrepancies were resolved through manual chart review with trained data abstractors.

### Outcome Variables

The outcomes of interest were receipt of maternal RSVpreF vaccine or infant nirsevimab. Infants were categorized into 1 of 4 mutually exclusive groups: maternal RSVpreF only, infant nirsevimab only, maternal RSVpreF and infant nirsevimab, or neither. Receipt of maternal RSVpreF and/or infant nirsevimab was based on the documented immunization and administration date recorded in the EHR and/or PIERS occurring during pregnancy or an infant's first RSV season, respectively. Because the objective of this study was to evaluate the uptake of RSV prevention strategies, group assignment was based on documented receipt of each product rather than the timing of administration relative to delivery. Accordingly, infants whose mothers received RSVpreF <14 days before delivery but did not receive nirsevimab remained classified as maternal RSVpreF only, whereas those who received both were classified in the maternal RSVpreF and infant nirsevimab group.

For descriptive analyses, nirsevimab receipt was categorized as per the age at administration (0-6 days, 7-30 days, and >30 days of life). However, all infants who received nirsevimab during their first RSV season were considered nirsevimab recipients in the primary multivariable analyses regardless of the age at administration. If an infant received more than 1 nirsevimab dose during their first RSV season, only the first dose was considered. Any discrepancies in documentation were resolved through manual EHR review by a physician. Infants who received an RSV vaccine or who were born to pregnant individuals who received a nonapproved RSV vaccine, whose RSV vaccine type could not be verified, or received RSVpreF in a previous pregnancy were excluded. Infants who received palivizumab were included and categorized based on the receipt of RSVpreF or nirsevimab. Dates were selected based on system-wide recommended seasonal RSV prevention product administration from the University of Pittsburgh Medical Center health system to clinicians during each season.

### Predictor Variables

We constructed a directed acyclic graph to represent hypothesized relationships between maternal, infant, and health system factors with maternal RSVpreF vaccination and infant nirsevimab receipt and to guide selection of covariates. Maternal and pregnancy characteristics included age (continuous), race and ethnicity (categorized by the investigators based on recorded race in the EHR at time of delivery), insurance type at delivery, area deprivation index (ADI), parity/sibling (number of living children), multifetal pregnancy, body mass index, medical comorbidities determined by *International Classification of Diseases*, *Tenth Revision*, coding (substance use, asthma, diabetes, chronic hypertension, hypertensive disorders of pregnancy, and other chronic conditions), history of preterm birth, adequacy of prenatal care (4 or more visits), suspected fetal congenital anomalies, and receipt of ≥1 other recommended vaccines during pregnancy (Tdap, influenza, or COVID-19). The ADI is a continuous (range, 1-100), validated socioeconomic measurement that reflects neighborhood income, education level, employment status, and quality of housing.^10^ To account for the timing of pregnancy relative to each RSV season, a continuous variable representing days from a season-specific reference date (season 1: January 1, 2023; season 2: January 1, 2024) to the estimated date of conception was constructed (conception days) using the date of delivery and documented gestational age at delivery.

Infant characteristics included race and ethnicity (categorized by the investigators based on recorded race in the EHR at the time of delivery), sex (biological sex assigned at birth), gestational age at birth, birth month, birth weight, congenital heart disease, chronic lung disease, receipt of a hepatitis B vaccine and vitamin K within 14 days of birth, and attendance at a well-child visit within 30 days of life.

### Statistical Analyses

The uptake of maternal RSVpreF vaccination and infant nirsevimab was summarized overall and by demographic, clinical, pregnancy-related, and vaccine coadministration characteristics across both seasons. Bivariate associations between variables identified as relevant in the directed acyclic graphs and uptake of each RSV-preventive product were evaluated using χ^2^ or Fisher exact tests for categorical variables and *t* tests for continuous variables. These variables were then included in separate multivariable models to estimate adjusted associations with RSVpreF and nirsevimab uptake; no data-driven variable selection procedures were used. Missing data were treated as a separate category. Comparisons among infants who received both RSVpreF and nirsevimab were not performed because of the small sample size.

To assess the stability of associations across seasons, season-specific multivariable models were constructed as sensitivity analyses using the same covariate set in the combined-season multivariable models. An additional sensitivity analysis was conducted after restricting the cohort to infants with ≥1 well-child visit within 30 days of birth within the health system using the same covariate set in the combined-season multivariable models.

All statistical analyses were performed using SAS, version 9.4 (SAS Institute).

## Results

From 13 374 infants, 10 196 were included in this study (Figure 1). Of those, 4555 were born from October 1, 2023, to April 15, 2024, (season 1), and 5641 were born from September 1, 2024, to April 30, 2025 (season 2). Overall, 5868/10 196(57.6%) infants who were born in-season had exposure to an RSV prevention product (Table I). This increased from 2100/4555 (46.1%) infants in season 1 to 3768/5641 (66.8%) infants in season 2 (Figures 2 and 3). The RSVpreF uptake increased from 1280/4555 (28.1%) to 2570/5641(45.6%) between seasons 1 and 2 (*P* < .001; Figures 2 and 3). Overall receipt of nirsevimab at any point in an infant's first RSV season increased from 749/4555 (16.4%) to 1088/5641 (19.3%) between seasons 1 and 2 (*P* < .001; Figures 2 and 3). Importantly, among infants who received nirsevimab, there was an increase in the proportion of infants who received it between 0 and 6 days of life between seasons 1 (242/749 [32.3%]) and season 2 (502/1088 [46.1%]; *P* < .001; eTable 1; available at www.jpeds.com). From 181/10 196 (1.8%) infants who received both RSVpreF and nirsevimab, 106/181 (58.6%) were exposed to maternal RSVpreF <14 days before delivery (eTable 1). Among nirsevimab recipients, 60.4% were identified in both EHR and PIERS, 24.0% in the EHR only, and 15.6% in the PIERS only. Among RSVpreF recipients, 84.4% were identified in both sources, 14.2% in the EHR only, and 1.4% in the PIERS only.

**Table I:** Descriptive characteristics of pregnant people and infants who received RSV prevention products during the 2023-2024 and 2024-2025 RSV seasons from a single health system

Characteristic	Maternal RSV preF			Nirsevimab			Neither§^,^¶		
	Overall	Season 1	Season 2	Overall	Season 1	Season 2	Overall	Season 1	Season 2
Maternal/pregnancy characteristics∗∗									
Age at delivery (y)									
Mean (SD)	31.4 (5.3)‡	31.4 (5.2)‡	31.3 (5.3)‡	30.5 (5.6)∗	30.6 (5.5)	30.4 (5.7)	30.1 (5.4)	30.1 (5.4)	30.2 (5.3)
Race††									
Asian	235 (6.1)‡	83 (6.5)‡	152 (5.9)‡	98 (5.3)†	38 (5.1)	60 (5.5)†	158 (3.7)	102 (4.2)	56 (3.0)
Black	389 (10.1)‡	129 (10.1)‡	260 (10.1)‡	330 (18.0)†	134 (17.9)	196 (18.0)†	838 (19.4)	495 (20.2)	343 (18.3)
White	2906 (75.5)‡	967 (75.5)‡	1939 (75.4)‡	1259 (68.5)†	516 (68.9)	743 (68.3)†	3017 (69.7)	1672 (68.1)	1345 (71.8)
Other	108 (2.8)‡	37 (2.9)‡	71 (2.8)‡	64 (3.5)†	18 (2.4)	46 (4.2)†	113 (2.6)	66 (2.7)	47 (2.5)
Declined/unknown	212 (5.5)‡	64 (5.0)‡	148 (5.8)‡	86 (4.7)†	43 (5.7)	43 (4.0)†	202 (4.7)	120 (4.9)	82 (4.4)
Ethnicity‡‡									
Hispanic or Latino	100 (2.6)‡	34 (2.7)	66 (2.6)†	65 (3.5)	24 (3.2)	41 (3.8)	126 (2.9)	74 (3.0)	52 (2.8)
Not Hispanic or Latino	3389 (88.0)‡	1152 (90.0)	2237 (87.0)†	1631 (88.8)	670 (89.5)	961 (88.3)	3910 (90.3)	2228 (90.8)	1682 (89.8)
Declined/unknown	361 (9.4)‡	94 (7.3)	267 (10.4)†	141 (7.7)	55 (7.3)	86 (7.9)	292 (6.7)	153 (6.2)	139 (7.4)
Health insurance at delivery									
Medical assistance	897 (23.3)‡	292 (22.8)‡	605 (23.5)‡	632 (34.4)†	235 (31.4)‡	397 (36.5)	1677 (38.7)	976 (39.8)	701 (37.4)
Private	2860 (74.3)‡	968 (75.6)‡	1892 (73.6)‡	1159 (63.1)†	498 (66.5)‡	661 (60.8)	2502 (57.8)	1392 (56.7)	1110 (59.3)
Self-pay	55 (1.4)‡	13 (1.0)‡	42 (1.6)‡	27 (1.5)†	10 (1.3)‡	17 (1.6)	97 (2.2)	54 (2.2)	43 (2.3)
Unknown	38 (1.0)‡	7 (0.5)‡	31 (1.2)‡	19 (1.0)†	6 (0.8)‡	13 (1.2)	52 (1.2)	33 (1.3)	19 (1.0)
ADI quartile									
1st	1134 (29.5)‡	399 (31.2)‡	735 (28.6)‡	406 (22.1)†	182 (24.3)‡	224 (20.6)	819 (18.9)	495 (20.2)	324 (17.3)
2nd	935 (24.3)‡	312 (24.4)‡	623 (24.2)‡	451 (24.6)†	208 (27.8)‡	243 (22.3)	964 (22.3)	539 (22.0)	425 (22.7)
3rd	896 (23.3)‡	284 (22.2)‡	612 (23.8)‡	426 (23.2)†	159 (21.2)‡	267 (24.5)	1108 (25.6)	614 (25.0)	494 (26.4)
4th	644 (16.7)‡	210 (16.4)‡	434 (16.9)‡	438 (23.8)†	153 (20.4)‡	285 (26.2)	1152 (26.6)	656 (26.7)	496 (26.5)
Missing	241 (6.3)‡	75 (5.9)‡	166 (6.5)‡	116 (6.3)†	47 (6.3)‡	69 (6.3)	285 (6.6)	151 (6.2)	134 (7.2)
Substance use									
No	3717 (96.5)†	1233 (96.3)	2484 (96.7)	1735 (94.4)	704 (94.0)	1031 (94.8)	4128 (95.4)	2339 (95.3)	1789 (95.5)
Yes	133 (3.5)†	47 (3.7)	86 (3.3)	102 (5.6)	45 (6.0)	57 (5.2)	200 (4.6)	116 (4.7)	84 (4.5)
BMI									
<30 kg/m^2^	2402 (62.4)	818 (63.9)	1584 (61.6)	1124 (61.2)	481 (64.2)	643 (59.1)	2691 (62.2)	1525 (62.1)	1166 (62.3)
≥30 kg/m^2^	1403 (36.4)	450 (35.2)	953 (37.1)	692 (37.7)	261 (34.8)	431 (39.6)	1587 (36.7)	902 (36.7)	685 (36.6)
Unknown/Missing	45 (1.2)	12 (0.9)	33 (1.3)	21 (1.1)	7 (0.9)	14 (1.3)	50 (1.2)	28 (1.1)	22 (1.2)
Asthma									
No	3419 (88.8)	1148 (89.7)	2271 (88.4)	1619 (88.1)	668 (89.2)	951 (87.4)	3819 (88.2)	2166 (88.2)	1653 (88.3)
Yes	431 (11.2)	132 (10.3)	299 (11.6)	218 (11.9)	81 (10.8)	137 (12.6)	509 (11.8)	289 (11.8)	220 (11.7)
Diabetes									
No	3460 (89.9)	1144 (89.4)	2316 (90.1)	1606 (87.4)†	654 (87.3)∗	952 (87.5)	3886 (89.8)	2216 (90.3)	1670 (89.2)
Yes	390 (10.1)	136 (10.6)	5 (0.2)	231 (12.6)†	95 (12.7)∗	6 (0.6)	442 (10.2)	239 (9.7)	3 (0.2)
Chronic hypertension									
No	3511 (91.2)	1172 (91.6)	2339 (91.0)	1655 (90.1)	679 (90.7)	976 (89.7)	3911 (90.4)	2232 (90.9)	1679 (89.6)
Yes	339 (8.8)	108 (8.4)	231 (9.0)	182 (9.9)	70 (9.3)	112 (10.3)	417 (9.6)	223 (9.1)	194 (10.4)
Other comorbid conditions									
No	3556 (92.4)	1188 (92.8)	2368 (92.1)	1679 (91.4)∗	695 (92.8)	984 (90.4)†	4022 (92.9)	2270 (92.5)	1752 (93.5)
Yes	294 (7.6)	92 (7.2)	202 (7.9)	158 (8.6)∗	54 (7.2)	104 (9.6)†	306 (7.1)	185 (7.5)	121 (6.5)
Hypertensive disorders of pregnancy									
No	2953 (76.7)^ND^	1028 (80.3)^ND^	1925 (74.9)^ND^	1325 (72.1)†	561 (74.9)∗	764 (70.2)	3299 (76.2)	1946 (79.3)	1353 (72.2)
Yes	897 (23.3)^ND^	252 (19.7)^ND^	645 (25.1)^ND^	512 (27.9)†	188 (25.1)∗	324 (29.8)	1029 (23.8)	509 (20.7)	520 (27.8)
History of preterm birth									
No	3760 (97.7)‡	1250 (97.7)∗	2510 (97.7)‡	1768 (96.2)^ND^	723 (96.5)^ND^	1045 (96.0)^ND^	4153 (96.0)	2359 (96.1)	1794 (95.8)
Yes	90 (2.3)‡	30 (2.3)∗	60 (2.3)‡	69 (3.8)^ND^	26 (3.5)^ND^	43 (4.0)^ND^	175 (4.0)	96 (3.9)	79 (4.2)
Parity/sibling									
0	1970 (51.2)‡	660 (51.6)‡	1310 (51.0)‡	855 (46.5)‡	356 (47.5)∗	499 (45.9)‡	1780 (41.1)	1060 (43.2)	720 (38.4)
≥1	1880 (48.8)‡	620 (48.4)‡	1260 (49.0)‡	982 (53.5)‡	393 (52.5)∗	589 (54.1)‡	2548 (58.9)	1395 (56.8)	1153 (61.6)
Prenatal care visits									
1-3 visits	7 (0.2)‡	3 (0.2)‡	4 (0.2)‡	52 (2.8)	16 (2.1)	36 (3.3)	118 (2.7)	75 (3.1)	43 (2.3)
≥4 visits	3843 (99.8)‡	1277 (99.8)‡	2566 (99.8)‡	1785 (97.2)	733 (97.9)	1052 (96.7)	4210 (97.3)	2380 (96.9)	1830 (97.7)
Multifetal gestation									
No	3763 (97.7)‡	1252 (97.8)∗	2511 (97.7)†	1730 (94.2)†	708 (94.5)∗	1022 (93.9)∗	4161 (96.1)	2365 (96.3)	1796 (95.9)
Yes	87 (2.3)‡	28 (2.2)∗	59 (2.3)†	107 (5.8)†	41 (5.5)∗	66 (6.1)∗	167 (3.9)	90 (3.7)	77 (4.1)
Suspected fetal congenital anomalies§§									
No	3204 (83.2)	1068 (83.4)	2136 (83.1)	1522 (82.9)	631 (84.2)	891 (81.9)	3597 (83.1)	2050 (83.5)	1547 (82.6)
Yes	646 (16.8)	212 (16.6)	434 (16.9)	315 (17.1)	118 (15.8)	197 (18.1)	731 (16.9)	405 (16.5)	326 (17.4)
Other recommended vaccine during pregnancy									
0	69 (1.8)‡	19 (1.5)‡	50 (1.9)‡	246 (13.4)‡	75 (10.0)‡	171 (15.7)‡	1204 (27.8)	592 (24.1)	612 (32.7)
≥1	3781 (98.2)‡	1261 (98.5)‡	2520 (98.1)‡	1591 (86.6)‡	674 (90.0)‡	917 (84.3)‡	3124 (72.2)	1863 (75.9)	1261 (67.3)
Conception days¶¶									
Mean (SD)	103.4 (55.4)	111.3 (34.5)∗	99.50 (62.94)‡	115.4 (100.2)^ND^	82.8 (52.0)^ND^	137.86 (117.71)^ND^	128.2 (72.9)	115.1 (59.9)	145.43 (84.01)
Infant characteristics									
Gestational age at birth (wk)									
<37^0/7^ wk	203 (5.3)^ND^	61 (4.8)^ND^	142 (5.6)^ND^	315 (17.1)‡	122 (16.3)†	193 (17.7)‡	512 (11.8)	364 (11.2)	238 (12.7)
≥37^0/7^ wk	3647 (94.7)^ND^	1219 (95.2)^ND^	2428 (94.5)^ND^	1522 (82.9)‡	627 (83.7)†	895 (82.3)‡	3816 (88.2)	2181 (88.8)	1635 (87.3)
Month of birth									
September	46 (1.2)^ND^	0 (0.0)^ND^	46 (1.8)^ND^	205 (11.2)‡	0 (0.0)‡	205 (18.8)‡	118 (2.7)	0 (0.0)	118 (6.3)
October	373 (9.7)^ND^	7 (0.5)^ND^	366 (14.2)^ND^	371 (20.2)‡	134 (17.9)‡	237 (21.8)‡	462 (10.7)	272 (11.1)	190 (10.1)
November	527 (13.7)^ND^	99 (7.7)^ND^	428 (16.7)^ND^	353 (19.2)‡	223 (29.8)‡	130 (11.9)‡	558 (12.9)	388 (15.8)	170 (9.1)
December	748 (19.4)^ND^	294 (23.0)^ND^	454 (17.7)^ND^	257 (14.0)‡	114 (15.2)‡	143 (13.1)‡	478 (11.0)	296 (12.1)	182 (9.7)
January	804 (20.9)^ND^	362 (28.3)^ND^	442 (17.2)^ND^	202 (11.0)‡	95 (12.7)‡	107 (9.8)‡	511 (11.8)	319 (13.0)	192 (10.3)
February	699 (18.2)^ND^	339 (26.5)^ND^	360 (14.0)^ND^	190 (10.3)‡	87 (11.6)‡	103 (9.5)‡	488 (11.3)	289 (11.8)	199 (10.6)
March	524 (13.6)^ND^	166 (13.0)^ND^	358 (13.9)^ND^	174 (9.5)‡	88 (11.7)‡	86 (7.9)‡	872 (20.1)	539 (22.0)	333 (17.8)
April	129 (3.4)^ND^	13 (1.0)^ND^	116 (4.5)^ND^	85 (4.6)‡	8 (1.1)‡	77 (7.1)‡	841 (19.4)	352 (14.3)	489 (26.1)
Received hepatitis B vaccination <14 d of life									
No	144 (3.7)^ND^	30 (2.3)^ND^	114 (4.4)^ND^	140 (7.6)‡	48 (6.4)‡	92 (8.5)‡	948 (21.9)	391 (15.9)	557 (29.7)
Yes	3706 (96.3)^ND^	1250 (97.7)^ND^	2456 (95.6)^ND^	1697 (92.4)‡	701 (93.6)‡	996 (91.5)‡	3380 (78.1)	2064 (84.1)	1316 (70.3)
Received intramuscular vitamin K < 14 d of life									
No	5 (0.1)^ND^	0 (0.0)^ND^	5 (0.2)^ND^	8 (0.4)‡	5 (0.7)†	3 (0.3)	137 (3.2)	52 (2.1)	85 (4.5)‡
Yes	3845 (99.9)^ND^	1280 (100.0)^ND^	2565 (99.8)^ND^	1829 (99.6)‡	744 (99.3)†	1085 (99.7)	4191 (96.8)	2403 (97.9)	1788 (95.5)‡
Well-child visit <30 d of life									
No	892 (23.2)^ND^	304 (23.8)^ND^	588 (22.9)^ND^	366 (19.9)‡	135 (18.0)‡	231 (21.2)‡	1707 (39.4)	879 (35.8)	828 (44.2)
Yes	2958 (76.8)^ND^	976 (76.2)^ND^	1982 (77.1)^ND^	1471 (80.1)‡	614 (82.0)‡	857 (78.8)‡	2621 (60.6)	1576 (64.2)	1045 (55.8)
Congenital heart disease									
No	3700 (96.1)^ND^	1230 (96.1)^ND^	2470 (96.1)^ND^	1749 (95.2)	720 (96.1)	1029 (94.6)	4123 (95.3)	2359 (96.1)	1764 (94.2)
Yes	150 (3.9)^ND^	50 (3.9)^ND^	100 (3.9)^ND^	88 (4.8)	29 (3.9)	59 (5.4)	205 (4.7)	96 (3.9)	109 (5.8)
Chronic lung disease									
No	3839 (99.7)^ND^	1276 (99.7)^ND^	2563 (99.7)^ND^	1835 (99.9)	748 (99.9)	1087 (99.9)	4317 (99.7)	2449 (99.8)	1868 (99.7)
Yes	11 (0.3)^ND^	4 (0.3)^ND^	7 (0.3)^ND^	2 (0.1)	1 (0.1)	1 (0.1)	11 (0.3)	6 (0.2)	5 (0.3)
Receipt of palivizumab									
No	3850 (100.0)^ND^	1280 (100.0)^ND^	2570 (100.0)^ND^	1834 (99.8)^ND^	746 (99.6)^ND^	1088 (100.0)^ND^	4324 (99.9)	2451 (99.8)	1873 (100.0)
Yes	0 (0.0)^ND^	0 (0.0)^ND^	0 (0.0)^ND^	3 (0.2)^ND^	3 (0.4)^ND^	0 (0.0)^ND^	4 (0.1)	4 (0.2)	0 (0.0)

*BMI*, body mass index; *ND*, not done.

∗ Significant at the 0.05 level when compared with the “neither” group.

† Significant at the 0.01 level when compared with the “neither” group.

‡ Significant at the <.001 level when compared with the “neither” group.

§ Comparisons were only completed for predictor variables included in the directed acyclic graph for RSVpreF and nirsevimab.

¶ N = 181 participants were exposed to both maternal RSVpreF and nirsevimab. Comparisons for this group were not carried out, and they were not included in the maternal RSVpreF-only or nirseivmab-only groups. Notably, N = 20 (11%) of these were multifetal gestations and N = 74 (40.9%) had a hypertensive disorder of pregnancy.

∗∗ (%) unless otherwise specified.

†† Race was categorized by the investigators based on recorded race in the EHR at the time of delivery. Asian includes “Asian Indian,” “other Asian,” “Vietnamese,” “Chinese,” and “Korean”; Black includes “Black/African American”; White includes “White”; other includes “other Pacific Islander” and “other”; unknown includes “unknown,” “unreported, chose not to disclose race.”

‡‡ Ethnicity was categorized by the investigators based on recorded ethnicity in the EHR at the time of delivery. Hispanic includes “another Hispanic, Latino/a or Spanish origin”, “Mexican, Mexican American, Chicano/a”, “Puerto Rican”; non-Hispanic includes “non-Hispanic or Latino/a”; unknown includes “not specified” or “unreported/chose not to disclose”.

§§ Suspected fetal congenital anomalies determined by *International Classification of Diseases, 10 Revision*, codes during pregnancy.

¶¶ Conception days were a continuous variable defined as days from January 1 of the corresponding year to the estimated date of conception based on the birth date and documented gestational age at delivery.

**Figure 2:**
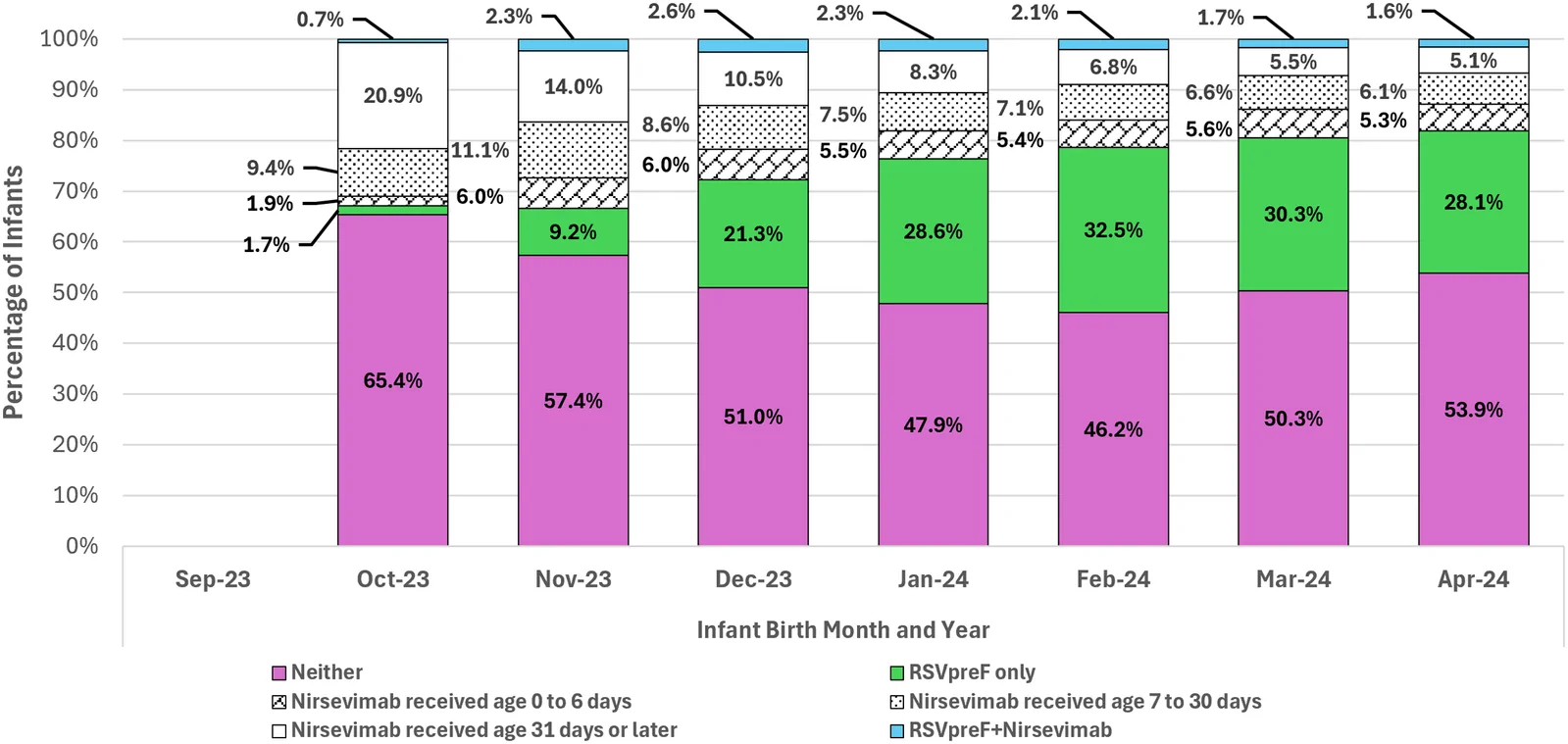
Cumulative uptake of maternal RSVpreF and infant nirsevimab, season 1. Cumulative proportion of newborns during the study period who were protected against RSV through bivalent RSVpreF vaccination, infant nirsevimab (shown by age in days at receipt), both, or neither during the 2023-2024 RSV season (season 1) Percentages not shown for values less than 6%.

**Figure 3:**
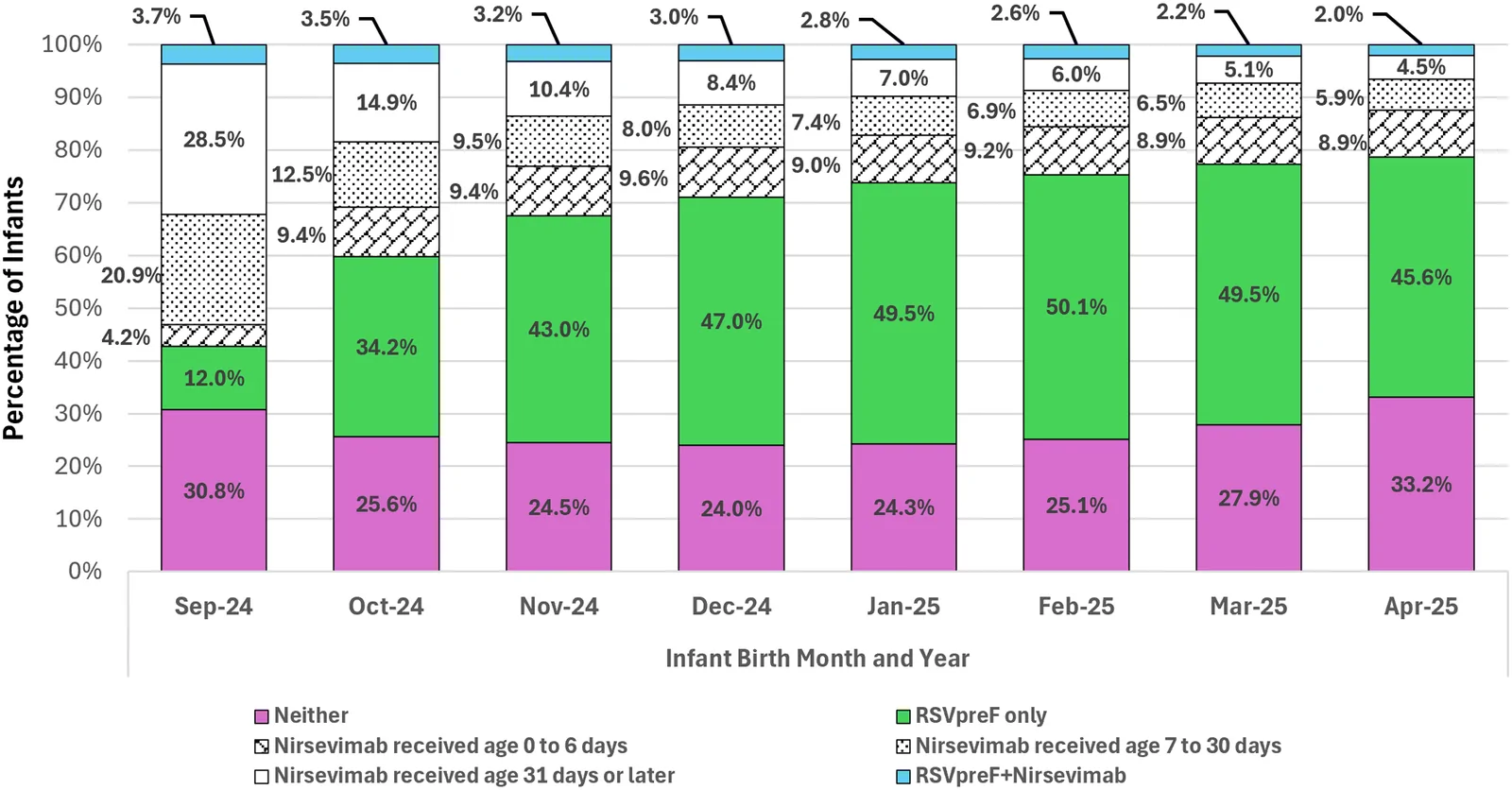
Cumulative uptake of maternal RSVpreF and infant nirsevimab, season 2. Cumulative proportion of newborns during the study period who were protected against RSV through bivalent RSVpreF vaccination, infant nirsevimab (shown by age in days at receipt), both, or neither during the 2024-2025 RSV season (season 2). Percentages not shown for values less than 6%.

### Bivariate Analyses

In combined-season 1 and 2 analyses, the uptake of both RSVpreF and nirsevimab was higher among individuals with markers of greater health care access and engagement, including older age, private insurance, lower ADI, and receipt of other recommended vaccines during pregnancy (Table I). RSVpreF uptake was additionally associated with receipt of more prenatal care visits and absence of prior preterm birth, whereas nirsevimab uptake was more strongly associated with infant clinical risk factors, including prematurity and receipt of routine preventative newborn care (vitamin K or hepatitis B vaccination).

Although overall patterns of RSVpreF uptake were similar across seasons, several shifts in nirsevimab uptake were observed over time. Between seasons, nirsevimab uptake increased among infants with a higher ADI and those with medical assistance (Table I). The uptake of either RSV-preventive product increased substantially among infants whose birth parents received other recommended vaccines during pregnancy (51.8%-73.7%, a 42.3% increase; Figure 4A) and among infants who received routine newborn preventive care (46.6%-67.8%, a 45.5% increase; Figure 4B). Importantly, nirsevimab uptake nearly doubled among infants born to women who received no other recommended vaccines (10.9%-20.5%; Figure 4A).

**Figure 4:**
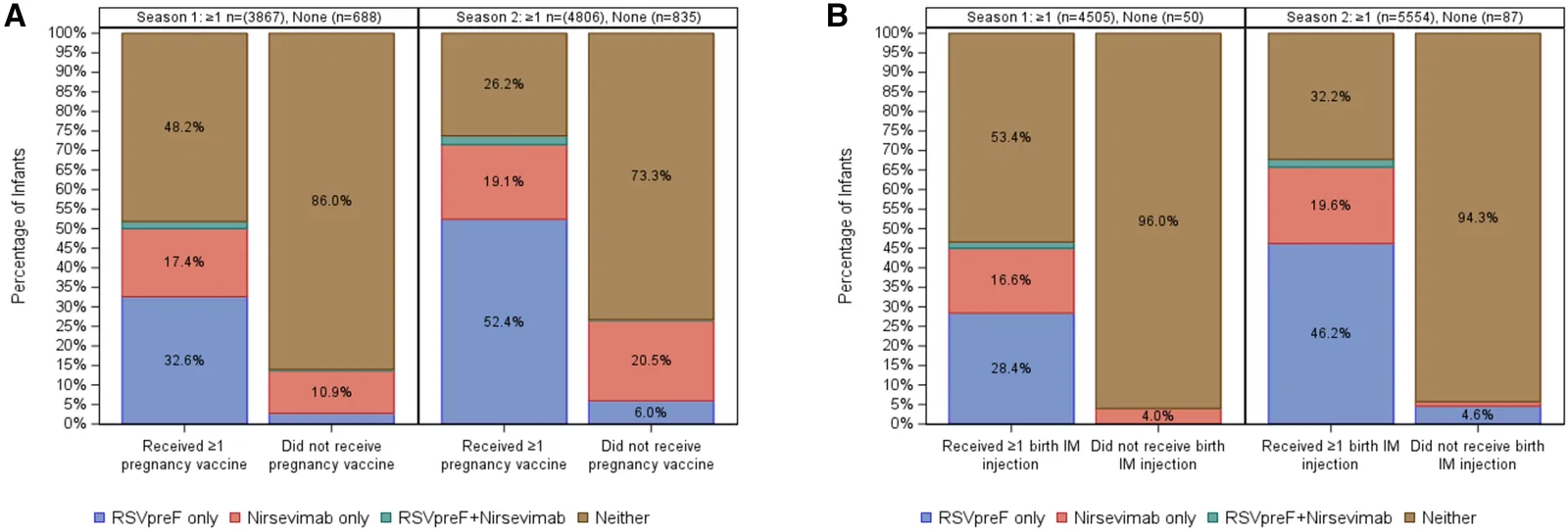
Uptake of maternal RSVpreF and infant nirsevimab by receipt of other recommended pregnancy vaccines and infant hepatitis B vaccine and vitamin K by season. **A**, Proportion of infants exposed to maternal bivalent RSVpreF vaccine, nirsevimab, or both stratified by maternal receipt of 1 or more recommended pregnancy vaccines (influenza, COVID-19, and Tdap) and RSV season. **B**, Proportion of infants exposed to maternal RSVpreF vaccine, nirsevimab, or both stratified by infant receipt of intramuscular hepatitis B vaccine or vitamin K within 14 days of birth and RSV season. Percentages not shown for values less than 2.5%.

### Multivariable Analyses

After multivariable adjustment, RSVpreF uptake was more likely among pregnant individuals with older age, Asian race (than with White race), private insurance (than with medical assistance), more prenatal care, and receipt of other recommended vaccines during pregnancy (Table II). A lower uptake was observed among individuals identified as Black race (than among White race), with a higher ADI, with a prior preterm birth, and with multifetal gestation and those with other children.

**Table II:** Multivariable model results of characteristics associated with RSV product uptake

Predictor variables∗^,^†	Maternal RSVpreF uptake vs neither RSV prevention product aOR (95% CI)	Infant nirsevimab uptake vs neither RSV prevention product aOR (95% CI)
Age at delivery (y)	1.03 (1.02-1.04)	1.01 (1.00-1.02)
Maternal race		
White	1 [reference]	1 [reference]
Black	0.75 (0.64-0.88)	0.99 (0.83-1.19)
Asian	1.28 (1.02-1.61)	1.33 (0.99-1.78)
Other	1.17 (0.85-1.62)	1.33 (0.92-1.92)
Declined/unknown	1.08 (0.85-1.38)	1.05 (0.77-1.43)
Maternal ethnicity		
Not Hispanic or Latino	1 [reference]	1 [reference]
Hispanic or Latino	0.83 (0.61-1.12)	1.05 (0.74-1.50)
Declined/unspecified	1.42 (1.16-1.73)	1.17 (0.91-1.50)
Health insurance at delivery		
Medical assistance	1 [reference]	1 [reference]
Private	1.40 (1.23-1.59)	1.20 (1.03-1.40)
Self-pay	1.02 (1.23-1.59)	0.87 (0.53-1.42)
Unknown/missing	1.25 (0.51-3.03)	0.72 (0.29-1.81)
ADI quartile		
First	1 [reference]	1 [reference]
Second	0.79 (0.68-0.91)	1.02 (0.85-1.23)
Third	0.77 (0.66-0.89)	0.91 (0.75-1.10)
Fourth	0.67 (0.57-0.80)	0.88 (0.71-1.08)
Missing	0.85 (0.68-1.06)	1.11 (0.84-1.48)
Substance use		
Yes	1.02 (0.79-1.31)	1.57 (1.19-2.08) 0.877
BMI		
<30 kg/m^2^	1 [reference]	1 [reference]
≥30 kg/m^2^	1.03 (0.92-1.14)	1.01 (0.88-1.15)
Unknown/missing	1.28 (0.58-2.86)	1.41 (0.57-3.45)
Asthma		
Yes	1.13 (0.96-1.32)	1.01 (0.84-1.23)
Diabetes		
Yes	0.91 (0.77-1.07)	1.23 (1.01-1.50)
Chronic hypertension		
Yes	0.97 (0.81-1.15)	0.82 (0.66-1.03)
Other comorbid conditions		
Yes	1.12 (0.93-1.35)	1.32 (1.05-1.65)
History of preterm birth		
Yes	0.63 (0.48-0.84)	ND/NA
Preterm birth		
Yes	ND	1.64 (1.36-1.97)
Parity/sibling		
0	1 [reference]	1 [reference]
≥1	0.70 (0.63-0.78)	0.80 (0.70-0.91)
Prenatal care visits		
1-3 visits	1 [reference]	1 [reference]
≥4 visits	5.79 (2.56-13.12)	0.65 (0.44-0.96)
Multifetal gestation		
Yes	0.69 (0.52-0.92)	1.67 (1.24-2.25)
Suspected fetal congenital anomalies		
Yes	1.01 (0.89-1.15)	0.92 (0.77-1.11)
Other recommended vaccines during pregnancy		
0	1 [reference]	1 [reference]
≥1	19.52 (15.18-25.10)	1.81 (1.52-2.16)
Conception days		
Mean	0.99 (0.99-0.99)	ND/NA
Hypertensive disorders of pregnancy		
Yes	ND	1.13 (0.97-1.30)
Birth month		
September	ND/NA	1 [reference]
October	ND/NA	0.43 (0.33-0.58)
November	ND/NA	0.35 (0.26-0.46)
December	ND/NA	0.31 (0.23-0.41)
January	ND/NA	0.23 (0.17-0.31)
February	ND/NA	0.22 (0.17-0.30)
March	ND/NA	0.09 (0.07-0.13)
April	ND/NA	0.04 (0.03-0.06)
Received hepatitis B vaccination < 14 d of life		
Yes	ND/NA	2.66 (2.14-3.31)
Received vitamin K < 14 d of life		
Yes	ND/NA	2.17 (1.02-4.64)
Well-child visit <30 d of life		
Yes	ND/NA	2.54 (2.20-2.94)
Congenital heart disease		
Yes	ND/NA	1.19 (0.86-1.66)
Congenital lung disease		
Yes	ND/NA	0.63 (0.13-2.98)

*BMI*, body mass index; *ND/NA*, not done/not applicable.

∗ A separate directed acyclic graph was used to represent assumptions of the causal structure of variables related to maternal RSVpreF uptake and infant nirsevimab uptake. These were used to guide variable selection in each multivariable model.

† The reference group is a group not shown for dichotomous variables (eg, if “no” is not in table, it is the reference group).

In contrast, nirsevimab uptake was independently associated with both maternal and infant factors, including maternal substance use, diabetes and other comorbidities, preterm birth, multifetal gestation, and receipt of other newborn preventatives (vitamin K and hepatitis B vaccination). Infants born later in the RSV season, those with siblings, and those without early well-child care within the health system were less likely to receive nirsevimab.

### Sensitivity Analyses

Sensitivity analysis of RSVpreF and nirsevimab uptake including only those with well-child care within the health care system in the first 30 days was mostly similar to the main analysis, strengthening many associations (eTable 2; available at www.jpeds.com).

Sensitivity analysis of RSVpreF uptake within each season demonstrated similar associations with differences in age, race, ADI, and having other children persisting in both season 1 and season 2 (eTable 3; available at www.jpeds.com). Conversely, sensitivity analysis of factors associated with nirsevimab uptake within each season demonstrated a loss of association between certain sociodemographic factors such as having private insurance and higher ADI from season 1 to season 2, whereas new associations emerged including increased odds of uptake if there was a multifetal gestation and decreased odds of uptake if there were siblings (eTable 3).

## Discussion

In this 2-season evaluation of RSV prevention strategies within a large integrated health system, the uptake of RSVpreF vaccination and nirsevimab increased substantially over time. However, nearly 40% of infants born during the second RSV season remain unprotected. The uptake of both products during the first season (46.1%) aligned with 2023-2024 RSV product uptake reported across 33 states, ranging between 11% and 53%.^5^ This lower first season uptake likely reflects early implementation challenges, including evolving national recommendations, product availability, and health system implementation during the initial rollout.^4^ Other barriers may have included insurance reimbursement and limited clinician and patient familiarity but still reflect remarkable acceptance for a first-season rollout. Although national data for the 2024-2025 season remain limited, our RSVpreF uptake in the second season (45.6%) was comparable with RSVpreF uptake estimates nationally from the Vaccine Safety Datalink (38.5%).^11^ Similarly, Acker et al found increased acceptance of RSVpreF from season 1 to season 2 in a large hospital system in New York, New York (21.1%-36.2%), consistent with our findings of improved adoption over time.^12^ In contrast, our study identifies lower nirsevimab uptake during the 2024-2025 season (19.3%) than estimates from the Center for Disease Control and Prevention for Pennsylvania (35.5%) and from the study by Acker et al (39.1%).^13^ Importantly, both of these studies include data on children born both during and outside of RSV season, which limits comparability as these studies represent a mixture of children who could be exposed to either product (those born during the season) and those who are only eligible for nirsevimab (born outside of the season).

Across both seasons, the uptake of maternal RSVpreF vaccination exceeded the uptake of infant nirsevimab. This pattern is consistent with prior survey data, indicating greater willingness among pregnant individuals to accept vaccination for themselves than immunoprophylaxis for their infant.^14^ However, differences in uptake likely reflect not only attitudes but also pragmatic features of care delivery. Maternal vaccination occurs during routine prenatal care, whereas nirsevimab administration depends on postpartum hospital workflows, early pediatric encounters, and coordination across care settings. Individuals receptive to RSV prevention may therefore accept the first or most readily available intervention, contributing to a higher uptake of RSVpreF relative to nirsevimab. These findings underscore the importance of considering both attitudinal and health system factors when interpreting uptake patterns.

Sociodemographic factors associated with uptake were broadly consistent with prior studies, with notable differences between prevention strategies.^5^^,^^6^^,^^14^^,^^15^ Lower uptake of RSVpreF among medical assistance and higher-deprivation populations persisted after adjustment across both seasons, whereas these disparities were attenuated for nirsevimab by the second season. This divergence may reflect system-level supports such as the Vaccines for Children program and the ability to administer nirsevimab during the birth hospitalization, reducing financial and access barriers. Alternatively, it may suggest greater acceptance of infant immunoprophylaxis among populations with lower vaccine acceptance during pregnancy, highlighting the potential advantage of a complementary 2-product prevention strategy.^16^

An important and unexpected finding was a 20%-30% lower odds of uptake of both RSVpreF and nirsevimab among families with other children, despite the well-established increased risk of RSV exposure associated with siblings.^17^ Similarly, Blauvelt et al found that first-time parents were 84% more likely to receive the RSVpreF vaccine than parents with other children.^15^ This discordance between actual risk and preventive behavior may reflect differences in risk perception among experienced parents, skepticism in using a new prevention product they did not use previously, or variation in provider counseling. Regardless of the mechanism, families with older children represent a key target population for future educational and implementation efforts.

Although uptake increased markedly across seasons among families who accepted other routine preventive services—such as vaccination during pregnancy or newborn preventive care with vitamin K and hepatitis B vaccine—substantial gaps persisted. Nearly one-third of infants who either received other newborn preventative care or had a birth parent who accepted other pregnancy vaccines still did not receive RSV prevention during the 2024-2025 season. Although parental hesitancy toward newly introduced prevention products may contribute to incomplete uptake even in these groups,^14^ targeted efforts to increase parental awareness coupled with strong, consistent provider recommendations are strategies with potential to further improve coverage. Moreover, during both seasons, most infants receiving nirsevimab were immunized after the first week of life despite recommendations for administration shortly after birth.^2^ This is also reflected in first-season national data.^5^^,^^6^ These delays likely suggest ongoing challenges related to workflow integration, reimbursement, and coordination between inpatient and outpatient care during a period of highest vulnerability. Efforts to reduce these barriers and increase immunization during the first week of life should be pursued.

Several limitations warrant consideration. Although linkage with the Pennsylvania Immunization Electronic Registry substantially improved ascertainment of RSVpreF and nirsevimab administration, uptake may still be underestimated if doses administered outside both the health system and registry were not captured.

Residual confounding related to vaccine attitudes and provider recommendation practices is possible. Results from a single health system with recommended RSV product administration dates based on local circulation may not be fully generalizable to other locations although observed uptake closely mirrored national estimates.

In conclusion, this evaluation across 2 RSV seasons in a single health system, uptake of maternal RSVpreF vaccination and infant nirsevimab increased over time but substantial gaps in protection remain including among infants with siblings and who engage in other preventative care. Differences in uptake between RSVpreF and niresvimab may reflect both attitudinal and health system factors, including timing of care, access to preventive services, and coordination between prenatal and pediatric settings. Such differences may be overcome through strong provider recommendations and systems that support timely identification of eligible individuals. Coordinated prenatal and postnatal implementation strategies may be essential to maximizing equitable RSV prevention during early infancy.

## CRediT authorship contribution statement

**Jessica E. Kerr:** Writing – review & editing, Writing – original draft, Visualization, Validation, Project administration, Methodology, Investigation, Formal analysis, Data curation, Conceptualization. **Annaliese J. Calzadilla:** Writing – review & editing, Writing – original draft, Conceptualization. **Hui Liu:** Writing – review & editing, Visualization, Validation, Methodology, Investigation, Formal analysis, Conceptualization. **Anne-Marie Rick:** Writing – review & editing, Writing – original draft, Validation, Supervision, Project administration, Methodology, Investigation, Funding acquisition, Formal analysis, Data curation, Conceptualization.

## Declaration of Competing Interest

A-M.R. has received honoraria and advisory fees from Pfizer and receives funding to the institution for unrelated work from Pfizer and the National Institutes of Health. This work was supported by the Department of Biomedical Informatics grant support [National Institutes of Health and Clinical and Translational Sciences Institute at the University of Pittsburgh Grant Number UL1-TR-001857] and A-M.R.’s support through the National Institute of Allergy and Infectious Disease K23AI159399. This work was supported in part by G. Nicholas III and Dorothy B. Beckwith. The study had no external funder or sponsor. No organization or entity had any role in the design and conduct of the study; collection, management, analysis, or interpretation of the data; preparation, review, or approval of the article; or decision to submit the article for publication. No products were provided for this study. J.K., A.C., and H.L. have no conflicts of interest relevant to this article to disclose.

## Supplementary Data

eTables 1-3
