# Supplementary material for: Uptake of Respiratory Syncytial Virus Prefusion F Protein Vaccination during Pregnancy and nirsevimab among Infants over Two Seasons in a Single Health System
Source: J Pediatr Clin Pract. 2026 Sep 15;22:200241. doi: 10.1016/j.jpedcp.2026.200241 (PMC13683871; doi:10.1016/j.jpedcp.2026.200241)
Supplement: Supplementary file 1 [file mmc1.docx]

**Table of Contents**

[eTable 1. Description of respiratory syncytial virus (RSV) prevention product receipt by group and RSV season.	2](#_Toc227741530)

[eTable 2. Multivariable sensitivity model of characteristics associated with RSV product uptake versus neither among those with ≥1 prenatal care visit and well-childcare < 30 days of life within the healthcare system.	3](#_Toc227741531)

[eTable 3. Multivariable sensitivity model of characteristics associated with RSV product uptake versus neither by RSV season.	7](#_Toc227741532)

| **eTable 1.** Description of respiratory syncytial virus (RSV) prevention product receipt by group and RSV season. | | | | | | | | | |
| --- | --- | --- | --- | --- | --- | --- | --- | --- | --- |
|  | **RSVpreF** | | | **Nirsevimab** | | | **RSVpreF and**  **Nirsevimab** | | |
|  | **Season** **1**  **(n = 1280)** | **Season 2**  **(n = 2570)** | **P*-*value** | **Season 1**  **(n = 749)** | **Season 2**  **(n = 1088)** | ***P* value** | **Season 1**  **(n = 71)** | **Season 2**  **(n = 110)** | **P*-*value** |
| **Characteristic** | | | | | | | | | |
| **Gestational age at maternal RSVpreF vaccination, week** | | | | | | | | | |
| Mean (SD) | 34.2 (1.5) | 34.0 (1.4) | <.001 | NA | NA | NA | 34.9 (1.6) | 34.6 (1.5) | 0.141 |
| <32^0/7^ | 13 (1.0) | 16 (0.6) | 0.345^‡^ | NA | NA | NA | 0 (0.0) | 2 (1.8) | 0.279^‡^ |
| 32^0/7^ to 36^6/7^ | 1262 (98.6) | 2540 (98.8) |  | NA | NA |  | 66 (93.0) | 105 (95.5) |  |
| ≥37^0/7^ | 5 (0.4) | 14 (0.5) |  | NA | NA |  | 5 (7.0) | 3 (2.7) |  |
| **Days from maternal RSVpreF vaccination to birth** | | | | | | | | | |
| <14 days | 54 (4.2) | 64 (2.5) | 0.003 | NA | NA | NA | 41 (57.7) | 65 (59.1) | 0.858 |
| ≥14 days | 1226 (95.8) | 2506 (97.5) |  | NA | NA |  | 30 (42.3) | 45 (40.9) |  |
| **Days after birth infant received nirsevimab** | | | | | | | | | |
| Mean (SD) | NA | NA | NA | 22.2 (24.6) | 17.3 (21.6) | <0.001 | 20.5 (20.1) | 20.5 (24.0) | 0.998 |
| 0 to 6 days | NA | NA | NA | 242 (32.3) | 502 (46.1) | <0.001 | 23 (32.4) | 40 (36.4) | 0.662 |
| 7 to 30 days | NA | NA |  | 276 (36.8) | 334 (30.7) |  | 27 (38.0) | 44 (40.0) |  |
| ≥31 days | NA | NA |  | 231 (30.8) | 252 (23.2) |  | 21 (29.6) | 26 (23.6) |  |
| ‡ Fisher’s Exact test. | | | | | | | | | |
| Abbreviations: RSVpreF: bivalent respiratory syncytial virus prefusion F protein; SD: standard deviation; NA: not applicable. | | | | | | | | | |

| **eTable 2.** Multivariable sensitivity model of characteristics associated with RSV product uptake versus neither among those with ≥1 prenatal care visit and well-childcare < 30 days of life within the healthcare system. | | | | |
| --- | --- | --- | --- | --- |
| **Predictor Variable^a^** | **Maternal RSVpreF uptake versus neither RSV prevention product** | | **Infant nirsevimab uptake versus neither RSV prevention product** | |
|  | **aOR (95% CI)** | **P-Value** | **aOR (95% CI)** | **P-Value** |
| **Age at delivery (years)** | | | | |
|  | 1.04 (1.02 - 1.05) | <.001 | 1.02 (1.00 - 1.03) | 0.052 |
| **Maternal race** | | | | |
| White | 1 [Reference] | 0.001 | 1 [Reference] | 0.477 |
| Black | 0.69 (0.57 - 0.84) |  | 1.03 (0.84 - 1.27) |  |
| Asian | 1.20 (0.91 - 1.57) |  | 1.23 (0.87 - 1.74) |  |
| Other | 1.13 (0.76 - 1.67) |  | 1.35 (0.88 - 2.07) |  |
| Declined / Unknown | 1.04 (0.77 - 1.41) |  | 1.18 (0.83 - 1.68) |  |
| **Maternal ethnicity** | | | | |
| Not Hispanic or Latino | 1 [Reference] | 0.002 | 1 [Reference] | 0.463 |
| Hispanic or Latino | 0.88 (0.62 - 1.26) |  | 1.11 (0.74 - 1.65) |  |
| Declined / Unspecified | 1.51 (1.19 - 1.93) |  | 1.19 (0.89 - 1.60) |  |
| **Health insurance at delivery** | | | | |
| Medical assistance | 1 [Reference] | <.001 | 1 [Reference] | 0.181 |
| Private | 1.41 (1.20 - 1.65) |  | 1.21 (1.01 - 1.45) |  |
| Self-pay | 1.03 (0.62 - 1.70) |  | 0.92 (0.51 - 1.68) |  |
| Unknown / Missing | 1.46 (0.51 - 4.15) |  | 1.21 (0.42 - 3.53) |  |
| **ADI quartile** | | | | |
| 1st | 1 [Reference] | 0.005 | 1 [Reference] | 0.157 |
| 2^nd^ | 0.76 (0.64 - 0.89) |  | 1.00 (0.81 - 1.23) |  |
| 3^rd^ | 0.77 (0.64 - 0.91) |  | 0.83 (0.66 - 1.03) |  |
| 4^th^ | 0.74 (0.60 - 0.90) |  | 0.86 (0.67 - 1.09) |  |
| Missing | 0.85 (0.65 - 1.12) |  | 1.13 (0.82 - 1.57) |  |
| **Substance use** | | | | |
| No | 1 [Reference] | 0.625 | 1 [Reference] | 0.001 |
| Yes | 1.08 (0.79 - 1.48) |  | 1.75 (1.25 - 2.45) |  |
| **BMI** | | | | |
| <30 kg/m^2^ | 1 [Reference] | 0.978 | 1 [Reference] | 0.593 |
| ≥30 kg/m^2^ | 1.00 (0.88 - 1.14) |  | 1.08 (0.93 - 1.26) |  |
| Unknown/Missing | 1.10 (0.43 - 2.85) |  | 1.22 (0.44 - 3.38) |  |
| **Asthma** | | | | |
| No | 1 [Reference] | 0.113 | 1 [Reference] | 0.879 |
| Yes | 1.17 (0.96 - 1.41) |  | 1.02 (0.82 - 1.27) |  |
| **Diabetes** | | | | |
| No | 1 [Reference] | 0.781 | 1 [Reference] | 0.006 |
| Yes | 0.97 (0.79 - 1.20) |  | 1.39 (1.10 - 1.76) |  |
| **Chronic hypertension** | | | | |
| No | 1 [Reference] | 0.684 | 1 [Reference] | 0.042 |
| Yes | 1.05 (0.85 - 1.29) |  | 0.77 (0.59 - 0.99) |  |
| **Other comorbid conditions** | | | | |
| No | 1 [Reference] | 0.381 | 1 [Reference] | 0.031 |
| Yes | 1.11 (0.88 - 1.41) |  | 1.35 (1.03 - 1.76) |  |
| **History of preterm birth** | | | | |
| No | 1 [Reference] | <.001 | ND | ND |
| Yes | 0.51 (0.35 - 0.73) |  | ND |  |
| **Preterm birth** | | | | |
| No | ND | ND | 1 [Reference] | 0.001 |
| Yes | ND |  | 1.47 (1.18 - 1.84) |  |
| **Parity/Sibling** | | | | |
| 0 | 1 [Reference] | <.001 | 1 [Reference] | 0.002 |
| ≥1 | 0.63 (0.56 - 0.72) |  | 0.78 (0.67 - 0.91) |  |
| **Prenatal care visits** | | | | |
| 1 to 3 visits | 1 [Reference] | 0.001 | 1 [Reference] | 0.129 |
| ≥4 visits | 4.97 (1.87 - 13.2) |  | 0.70 (0.44 - 1.11) |  |
| **Multi-fetal gestation** | | | | |
| No | 1 [Reference] | 0.181 | 1 [Reference] | 0.003 |
| Yes | 0.78 (0.55 - 1.12) |  | 1.72 (1.20 - 2.47) |  |
| **Fetal congenital anomalies** | | | | |
| No | 1 [Reference] | 0.836 | 1 [Reference] | 0.806 |
| Yes | 1.02 (0.87 - 1.19) |  | 0.97 (0.79 - 1.21) |  |
| **Other recommended vaccines during pregnancy** | | | | |
| 0 | 1 [Reference] | <.001 | 1 [Reference] | <.001 |
| ≥1 | 16.2 (11.9 - 22.2) |  | 1.58 (1.28 - 1.94) |  |
| **Conception days** | | | | |
| Mean | 0.99 (0.99 - 0.99) | <.001 | ND | ND |
| **Hypertensive disorders of pregnancy** | | | | |
| No | ND | ND | 1 [Reference] | 0.133 |
| Yes | ND |  | 1.14 (0.96 - 1.35) |  |
| **Birth Month** | | | | |
| September | ND | ND | 1 [Reference] | <.001 |
| October | ND |  | 0.36 (0.25 - 0.51) |  |
| November | ND |  | 0.30 (0.21 - 0.42) |  |
| December | ND |  | 0.23 (0.16 - 0.34) |  |
| January | ND |  | 0.19 (0.13 - 0.27) |  |
| February | ND |  | 0.19 (0.13 - 0.28) |  |
| March | ND |  | 0.08 (0.06 - 0.12) |  |
| April | ND |  | 0.04 (0.03 - 0.06) |  |
| **Received hepatitis B vaccination <14 days of life** | | | | |
| No | ND | ND | 1 [Reference] | <.001 |
| Yes | ND |  | 3.37 (2.52 - 4.51) |  |
| **Received vitamin K <14 days of life** | | | | |
| No | ND | ND | 1 [Reference] | 0.074 |
| Yes | ND |  | 2.74 (0.91 - 8.27) |  |
| **Congenital heart disease** | | | | |
| No | ND | ND | 1 [Reference] | 0.949 |
| Yes | ND |  | 0.99 (0.67 - 1.46) |  |
| **Congenital lung disease** | | | | |
| No | ND | ND | 1 [Reference] | 0.358 |
| Yes | ND |  | 2.37 (0.38 - 14.83) |  |
| ^a^A separate directed acyclic graph (DAG) was used to represent assumptions of the causal structure of variables related to maternal RSVpreF uptake and infant nirsevimab uptake. These were used to guide variable selection in each multivariable model. | | | | |
| **Abbreviations:** RSVpreF: bivalent respiratory syncytial virus prefusion F protein; aOR: adjusted odds ratio; CI: confidence interval; ADI: area deprivation index; BMI: Body mass index**;** ND: Not done | | | | |

| **eTable 3.** Multivariable sensitivity model of characteristics associated with RSV product uptake versus neither by RSV season. | | | | | | | | |
| --- | --- | --- | --- | --- | --- | --- | --- | --- |
| **Predictor variable^a^** | **Maternal RSVpreF uptake versus neither RSV prevention product** | | | | **Infant nirsevimab uptake versus neither RSV prevention product** | | | |
|  | **Season 1** | | **Season 2** | | **Season 1** | | **Season 2** | |
|  | **aOR (95% CI)** | **P-value** | **aOR (95% CI)** | **P-value** | **aOR (95% CI)** | **P-value** | **aOR (95% CI)** | **P-value** |
| **Age at delivery (years)** | | | | | | | | |
|  | 1.03 (1.01 - 1.04) | 0.001 | 1.04 (1.02 - 1.05) | <.001 | 1.01 (0.99 - 1.03) | 0.283 | 1.01 (0.99 - 1.03) | 0.319 |
| **Maternal race** | | | | | | | | |
| White | 1 [Reference] | 0.073 | 1 [ Reference] | 0.027 | 1 [ Reference] | 0.604 | 1 [Reference] | 0.233 |
| Black | 0.72 (0.56 - 0.92) |  | 0.83 (0.65 - 1.04) |  | 1.05 (0.80 - 1.37) |  | 1.01 (0.78 - 1.31) |  |
| Asian | 1.17 (0.85 - 1.62) |  | 1.48 (1.03 - 2.12) |  | 1.25 (0.82 - 1.93) |  | 1.42 (0.92 - 2.21) |  |
| Other | 1.10 (0.69 - 1.75) |  | 1.42 (0.87 - 2.31) |  | 1.05 (0.57 - 1.92) |  | 1.50 (0.90 - 2.51) |  |
| Declined / Unknown | 1.02 (0.72 - 1.45) |  | 1.27 (0.87 - 1.86) |  | 1.34 (0.88 - 2.06) |  | 0.85 (0.53 - 1.36) |  |
| **Maternal ethnicity** | | | | | | | | |
| Not Hispanic or Latino | 1 [Reference] | 0.214 | 1 [ Reference] | 0.095 | 1 [ Reference] | 0.484 | 1 [Reference] | 0.861 |
| Hispanic or Latino | 0.79 (0.51 - 1.25) |  | 0.84 (0.54 - 1.32) |  | 0.94 (0.55 - 1.60) |  | 1.14 (0.69 - 1.90) |  |
| Declined / Unspecified | 1.23 (0.90 - 1.67) |  | 1.33 (1.00 - 1.78) |  | 1.24 (0.86 - 1.79) |  | 1.05 (0.73 - 1.50) |  |
| **Health insurance at delivery** | | | | | | | | |
| Medical assistance | 1 [Reference] | 0.002 | 1 [ Reference] | 0.008 | 1 [ Reference] | 0.027 | 1 [Reference] | 0.441 |
| Private | 1.41 (1.15 - 1.72) |  | 1.38 (1.15 - 1.66) |  | 1.36 (1.08 - 1.71) |  | 1.11 (0.89 - 1.37) |  |
| Self-pay | 0.70 (0.36 - 1.38) |  | 1.15 (0.66 - 2.00) |  | 0.88 (0.41 - 1.86) |  | 0.90 (0.45 - 1.78) |  |
| Unknown / Missing | 0.73 (0.22 - 2.48) |  | 1.47 (0.22 - 10.0) |  | 0.51 (0.15 - 1.76) |  | 5.82 (0.39 - 86.1) |  |
| **ADI quartile** | | | | | | | | |
| 1st | 1 [Reference] | 0.031 | 1 [ Reference] | <.001 | 1 [ Reference] | 0.035 | 1 [Reference] | 0.749 |
| 2^nd^ | 0.84 (0.68 - 1.03) |  | 0.67 (0.54 - 0.83) |  | 1.11 (0.86 - 1.43) |  | 0.91 (0.69 - 1.20) |  |
| 3^rd^ | 0.74 (0.60 - 0.92) |  | 0.73 (0.58 - 0.90) |  | 0.79 (0.60 - 1.03) |  | 0.92 (0.70 - 1.22) |  |
| 4^th^ | 0.69 (0.54 - 0.89) |  | 0.59 (0.46 - 0.75) |  | 0.78 (0.57 - 1.06) |  | 0.84 (0.62 - 1.13) |  |
| Missing | 0.86 (0.61 - 1.20) |  | 0.73 (0.53 - 1.02) |  | 1.12 (0.74 - 1.69) |  | 1.04 (0.69 - 1.59) |  |
| **Substance use** | | | | | | | | |
| No | 1 [Reference] | 0.632 | 1 [ Reference] | 0.907 | 1 [ Reference] | 0.002 | 1 [Reference] | 0.085 |
| Yes | 1.10 (0.75 - 1.60) |  | 0.98 (0.68 - 1.41) |  | 1.91 (1.28 - 2.84) |  | 1.44 (0.95 - 2.16) |  |
| **BMI** | | | | | | | | |
| <30 kg/m^2^ | 1 [Reference] | 0.651 | 1 [ Reference] | 0.509 | 1 [ Reference] | 0.285 | 1 [Reference] | 0.227 |
| ≥30 kg/m^2^ | 0.96 (0.82 - 1.13) |  | 1.09 (0.94 - 1.28) |  | 0.87 (0.71 - 1.06) |  | 1.14 (0.94 - 1.38) |  |
| Unknown/Missing | 1.47 (0.56 - 3.88) |  | 1.32 (0.21 - 8.10) |  | 1.39 (0.44 - 4.45) |  | 0.24 (0.02 - 3.17) |  |
| **Asthma** | | | | | | | | |
| No | 1 [Reference] | 0.686 | 1 [ Reference] | 0.373 | 1 [ Reference] | 0.478 | 1 [Reference] | 0.58 |
| Yes | 1.05 (0.83 - 1.34) |  | 1.11 (0.89 - 1.39) |  | 0.90 (0.68 - 1.20) |  | 1.08 (0.82 - 1.41) |  |
| **Diabetes** | | | | | | | | |
| No | 1 [Reference] | 0.92 | 1 [ Reference] | 0.256 | 1 [ Reference] | 0.013 | 1 [Reference] | 0.508 |
| Yes | 0.99 (0.78 - 1.26) |  | 0.87 (0.68 - 1.11) |  | 1.44 (1.08 - 1.92) |  | 1.10 (0.83 - 1.47) |  |
| **Chronic hypertension** | | | | | | | | |
| No | 1 [Reference] | 0.782 | 1 [ Reference] | 0.603 | 1 [ Reference] | 0.603 | 1 [Reference] | 0.105 |
| Yes | 0.96 (0.74 - 1.26) |  | 0.94 (0.73 - 1.20) |  | 0.92 (0.66 - 1.27) |  | 0.78 (0.57 - 1.06) |  |
| **Other comorbid conditions** | | | | | | | | |
| No | 1 [Reference] | 0.694 | 1 [ Reference] | 0.056 | 1 [ Reference] | 0.831 | 1 [Reference] | 0.001 |
| Yes | 0.95 (0.71 - 1.25) |  | 1.32 (0.99 - 1.75) |  | 0.96 (0.68 - 1.36) |  | 1.76 (1.27 - 2.45) |  |
| **History of preterm birth** | | | | | | | | |
| No | 1 [Reference] | 0.098 | 1 [ Reference] | 0.015 | ND | ND | ND | ND |
| Yes | 0.69 (0.44 - 1.07) |  | 0.61 (0.41 - 0.91) |  | ND |  | ND |  |
| **Preterm birth** | | | | | | | | |
| No | ND | ND | ND | ND | 1 [ Reference] | <.001 | 1 [Reference] | <.001 |
| Yes | ND |  | ND |  | 1.66 (1.26 - 2.19) |  | 1.66 (1.27 - 2.16) |  |
| **Parity/Sibling** | | | | | | | | |
| 0 | 1 [Reference] | 0.001 | 1 [ Reference] | <.001 | 1 [ Reference] | 0.092 | 1 [Reference] | <.001 |
| ≥1 | 0.77 (0.66 - 0.90) |  | 0.61 (0.52 - 0.71) |  | 0.85 (0.70 - 1.03) |  | 0.66 (0.54 - 0.80) |  |
| **Prenatal care visits** | | | | | | | | |
| 1 to 3 visits | 1 [Reference] | 0.024 | 1 [ Reference] | <.001 | 1 [ Reference] | 0.267 | 1 [Reference] | 0.058 |
| ≥4 visits | 4.06 (1.21 - 13.6) |  | 9.50 (2.95 - 30.59) |  | 0.70 (0.38 - 1.31) |  | 0.59 (0.34 - 1.02) |  |
| **Multi-fetal gestation** | | | | | | | | |
| No | 1 [Reference] | 0.083 | 1 [Reference] | 0.05 | 1 [ Reference] | 0.061 | 1 [Reference] | 0.002 |
| Yes | 0.67 (0.42 - 1.05) |  | 0.67 (0.45 - 1.00) |  | 1.52 (0.98 - 2.36) |  | 1.96 (1.28 - 2.99) |  |
| **Fetal congenital anomalies** | | | | | | | | |
| No | 1 [Reference] | 0.612 | 1 [Reference] | 0.512 | 1 [ Reference] | 0.977 | 1 [Reference] | 0.708 |
| Yes | 1.05 (0.87 - 1.28) |  | 0.94 (0.78 - 1.13) |  | 0.99 (0.59 - 1.67) |  | 0.95 (0.73 - 1.24) |  |
| **Other recommended vaccines during pregnancy** | | | | | | | | |
| 0 | 1 [Reference] | <.001 | 1 [Reference] | <.001 | 1 [ Reference] | <.001 | 1 [Reference] | <.001 |
| ≥1 | 17.58 (11.0 - 28.0) |  | 27.5 (20.2 - 37.6) |  | 2.06 (1.53 - 2.76) |  | 1.92 (1.52 - 2.42) |  |
| **Conception days** | | | | | | | | |
| Mean | 1.00 (1.00 - 1.00) | 0.003 | 0.99 (0.99 - 0.99) | <.001 | ND | ND | ND | ND |
| **Hypertensive disorders of pregnancy** | | | | | | | | |
| No | ND | ND | ND | ND | 1 [ Reference] | 0.176 | 1 [Reference] | 0.903 |
| Yes | ND |  | ND |  | 1.16 (0.93 - 1.45) |  | 0.99 (0.80 - 1.22) |  |
| **Birth Month** | | | | | | | | |
| September | ND | ND | ND | ND | ND | <.001 | 1 [Reference] | <.001 |
| October | ND |  | ND |  | 1 [ Reference] |  | 0.78 (0.56 - 1.08) |  |
| November | ND |  | ND |  | 1.24 (0.93 - 1.64) |  | 0.51 (0.36 - 0.73) |  |
| December | ND |  | ND |  | 0.86 (0.63 - 1.18) |  | 0.55 (0.39 - 0.79) |  |
| January | ND |  | ND |  | 0.68 (0.49 - 0.95) |  | 0.36 (0.25 - 0.51) |  |
| February | ND |  | ND |  | 0.69 (0.50 - 0.97) |  | 0.32 (0.23 - 0.47) |  |
| March | ND |  | ND |  | 0.31 (0.22 - 0.43) |  | 0.13 (0.09 - 0.18) |  |
| April | ND |  | ND |  | 0.04 (0.02 - 0.08) |  | 0.07 (0.05 - 0.10) |  |
| **Received hepatitis B vaccination <14 days of life** | | | | | | | | |
| No | ND | ND | ND | ND | 1 [ Reference] | 0.001 | 1 [Reference] | <.001 |
| Yes | ND |  | ND |  | 1.88 (1.31 - 2.69) |  | 3.97 (3.00 - 5.25) |  |
| **Received vitamin K <14 days of life** | | | | | | | | |
| No | ND | ND | ND | ND | 1 [ Reference] | 0.996 | 1 [Reference] | 0.022 |
| Yes | ND |  | ND |  | 1.00 (0.36 - 2.77) |  | 4.16 (1.23 - 14.09) |  |
| **Congenital heart disease** | | | | | | | | |
| No | ND | ND | ND | ND | 1 [ Reference] | 0.977 | 1 [Reference] | 0.376 |
| Yes | ND |  | ND |  | 0.99 (0.59 - 1.67) |  | 1.23 (0.78 - 1.94) |  |
| **Congenital lung disease** | | | | | | | | |
| No | ND | ND | ND | ND | 1 [ Reference] | 0.961 | 1 [Reference] | 0.555 |
| Yes | ND |  | ND |  | 1.06 (0.12 – 9.29) |  | 0.50 (0.05 – 4.91) |  |
| ^a^A separate directed acyclic graph (DAG) was used to represent assumptions of the causal structure of variables related to maternal RSVpreF uptake and infant nirsevimab uptake. These were used to guide variable selection in each multivariable model. | | | | | | | | |
| **Abbreviations:** RSVpreF: bivalent respiratory syncytial virus prefusion F protein; aOR: adjusted odds ratio; CI: confidence interval; ADI: area deprivation index; BMI: Body mass index**;** ND: Not done | | | | | | | | |
